# Identification of kaempferol as an OSX upregulator by network pharmacology-based analysis of qianggu Capsule for osteoporosis

**DOI:** 10.3389/fphar.2022.1011561

**Published:** 2022-09-23

**Authors:** Ann Yehong Huang, Zhencheng Xiong, Kuankuan Liu, Yanan Chang, Li Shu, Guolan Gao, Chi Zhang

**Affiliations:** ^1^ Department of Biochemistry, University of Texas Southwestern Medical Center, Dallas, TX, United States; ^2^ Central Laboratory, Peking University International Hospital, Beijing, China; ^3^ Department of Obstetrics and Gynecology, Peking University International Hospital, Beijing, China; ^4^ Department of Orthopedics, Peking University International Hospital, Beijing, China; ^5^ Biomedical Engineering Department, Peking University, Beijing, China

**Keywords:** kaempferol, osteoporosis, qianggu capsule, gusuibu, OSX, network pharmacology

## Abstract

Osteoporosis is the most common metabolic disease of skeleton with reduced bone density and weaker bone. Qianggu Capsule as a traditional chinese medicine has been widely used to treat osteoporosis. The potential pharmacological mechanism of its active ingredient Gusuibu is not well understood. The purpose of this work is to analyze the anti-osteoporosis function of Gusuibu based on network pharmacology, and further explore the potential mechanism of Qianggu Capsule. The active compounds and their corresponding targets of Gusuibu were obtained from TCMSP, TCMID, and BATMAN-TCM databases. Potential therapeutic targets for osteoporosis were obtained through DisGeNET, TTD, GeneCards, MalaCards, CTD, and OMIM databases. The overlapping targets of Gusuibu and osteoporosis were obtained. GO and KEGG pathway enrichment analysis were performed. The “Gusuibu-active compounds-target genes-osteoporosis” network and protein-protein interaction (PPI) network were constructed, and the top hub genes were screened by using the plug-in CytoHubba. Molecular docking was used to verify the binding activity of hub genes and key compounds. We identified 21 active compounds and 140 potential therapeutic targets that may be related to Gusuibu and 10 hub genes (AKT1, IL6, JUN, TNF, MAPK3, VEGFA, EGFR, MAPK1, CASP3, PTGS2). Molecular docking analysis demonstrated that four key active small molecules in Gusuibu (including Luteolin, Naringenin, Kaempferol, and Beta-sitosterol) have excellent binding affinity to the target proteins encoded by the top 10 hub genes. Our new findings indicated that one key active compound kaempferol activated the expression of osteoblast specific transcription factor OSX through JNK kinase pathway.

## Introduction

Osteoporosis is the most common metabolic disease of skeleton with reduced bone density and weaker bone structure. Patients are more likely to have fractures with the pain and other complications, and the quality of life is dramatically affected (Zheng et al., 2020). Postmenopausal osteoporosis is one common form of primary osteoporosis. Osteoporosis is classified into the categories of “Gu Bi” and “Gu Lou” in the “Huang Di Nei Jing” of Traditional Chinese Medicine (TCM) theory (CSoOaBM, 2019). Bone metabolism is a continuous process, and bone mass will be gradually lost after the age of 35. People especially postmenopausal women and the elderly should pay more attention to osteoporosis prevention (Li et al., 2020a; Saul and Kosinsky, 2021). The fragility fractures induced by osteoporosis will lead to increased disability and mortality, resulting in a heavy family, social and economic burden (Kanis, 2002; Xiong et al., 2021). The commonly used anti-osteoporosis drugs include estrogen, calcium, vitamin D, bisphosphonates, and denosumab; however, the consequent adverse effects are still a challenge to be solved due to the increased duration of use and dose (Jackson and Mysiw, 2014). Therefore, it is needed to explore potential anti-osteoporosis drugs with high efficacy and safety.

It is well known that TCM has a long history of practice in China and other Asian countries to treat a wide range of diseases, including osteoporosis (Li et al., 2017a; Wei et al., 2017; Wu et al., 2017; Zhang et al., 2017; Ge et al., 2018; Li et al., 2020a; Li et al., 2020b; Liu et al., 2020; Zheng et al., 2020). Among those to treat osteoporosis, the clinically commonly used Traditional Chinese Medicine prescriptions include Qianggu Capsule, XianlingGubao Capsule, DuhuoJisheng Decoction, LiuweiDihuang Pill, and Erxian Decoction (Li et al., 2017a; Wei et al., 2017; Wu et al., 2017; Ge et al., 2018; Li et al., 2020a). Qianggu Capsule (Drug approval number: Z20030007, Qi-Huang Pharmaceutical CO. LTD., Beijing, China) is a China Food and Drug Administration (CFDA)-approved TCM for the treatment of osteoporosis and bone loss. The active ingredients in Qianggu Capsule are total flavonoids extracted from Rhizoma Drynariae (English name is Fortune’s Drynaria Rhizome, and Chinese name is Gusuibu), the dried rhizome of Drynaria fortune (Kunze) J. SM. (Wang et al., 2008; CSoOaBM, 2019). The active ingredient of Qianggu Capsules is the herbal Gusuibu. Gusuibu is still a kind of raw material, and there are many compounds in Gusuibu, including flavonoids. Studies have shown that the flavonoids in Gusuibu may treat osteoporosis by improving bone density and reducing bone loss (Zhang et al., 2017; Shen et al., 2020). However, the mechanism of function of many TCM cannot be elucidated due to the complexity of the ingredients in TCM.

In recent years, many researchers have started to use bioinformatics and network pharmacology to analyze the multi-component, multi-target and multi-pathway characteristics of TCM in order to elucidate mechanisms of its action, thus providing directions for further research (Shuai et al., 2020). Gusuibu contains a variety of components. Although some studies have suggested that total flavonoids play a key role in anti-osteoporosis (Wang et al., 2008; Zhang et al., 2017), the molecular mechanism has not been elucidated yet.

In this study we used a network pharmacology approach to analyze the key genes, active compounds and pathways in the anti-osteoporosis of Gusuibu, and further explored possible underlying molecular mechanisms. We identified one key active compound kaempferol in Gusuibu as an upregulator of osteoblast specific transcription factor OSX.

## Materials and methods

### Exploring potential pharmacodynamic compounds and relevant targets of gusuibu

Using TCM Systems Pharmacology (TCMSP, Version: 2.3, https://tcmspw.com/tcmsp.php) database (Ru et al., 2014), BATMAN-TCM platform (http://bionet.ncpsb.org/batman-tcm/) (Liu et al., 2016) and TCM Integrated Database (TCMID, http://www.megabionet.org/tcmid/) (Huang et al., 2018), the corresponding compounds of Gusuibu and related information were obtained. The compounds retrieved in the previous step were screened for active compounds according to the absorption, distribution, metabolism and excretion (ADME) protocol (oral bioavailability (OB) ≥ 30 and drug-likeness (DL) ≥ 0.18) (Tsaioun et al., 2016; Sheng et al., 2020). The databases were then used to mine potential targets corresponding to the active compounds. The target proteins were then converted to the corresponding gene names and UniProt ID using the UniProt database (https://www.uniprot.org/) for “*Homo sapiens*” (UniProt Consortium, 2018).

### Exploring the relevant targets of osteoporosis

The targets related to osteoporosis were obtained through retrieving GeneCards (https:// www.genecards.org/) (Relevance score ≥10) (Stelzer et al., 2016), MalaCards (https://www.malacards.org/) (Rappaport et al., 2017), DisGeNet database (https://www.disgenet.org/, v7.0) (Score ≥ 0.01) (Piñero et al., 2020), Therapeutic Target Database (TTD) (http://db.idrblab.net/ttd/, Last update by 1 June 2020) (Wang et al., 2020), Comparative Toxicogenomics Database (CTD) (http://ctdbase.org/, Last update by June 2020) (Inference score ≥ 15) (Davis et al., 2020), and Online Mendelian Inheritance in Man (OMIM) (https://omim.org/, updated 25 November 2020) (Amberger and Hamosh, 2017) using the keyword “osteoporosis or postmenopausal osteoporosis”. The potential targets obtained from the these 6 databases were integrated and deduplicated to generate the osteoporosis-related target set.

### Network construction and protein-protein interaction analysis

Gusuibu-related targets and osteoporosis-related targets were input to the Vennonline tool (http://www.bioinformatics.com.cn/) to obtain the common targets. These were the candidate targets of Gusuibu to treat osteoporosis. Cytoscape software (version 3.7.2) was used to construct the network diagram of “Gusuibu-active compounds-target genes-osteoporosis” (Shannon et al., 2003).

PPI is the basis of most biological processes and is important to understand cell physiology in both normal and disease states (Xiao et al., 2020). The STRING database (http://string-db.org/; version 11) was used to perform a PPI network analysis on the common targets (Szklarczyk et al., 2017). The species is limited to “*Homo sapiens*” with a confidence level greater than 0.4. Cytoscape software (version 3.7.2) was used to construct the PPI network, and the plug-in 12 CytoHubba algorithms [Degree, Maximal Clique Centrality (MCC), Clustering Coefficient, Density of Maximum Neighborhood Component (DMNC), BottleNeck, Maximum Neighborhood Component (MNC), Radiality, Edge Percolated Component (EPC), EcCentricity, Closeness, Betweenness, Stress] were used to find the top 10 hub genes (Chin et al., 2014; Xu et al., 2020).

### Enrichment analysis of GO and KEGG pathway

The cluster Profiler package in R (R 4.0.2 for Windows) was used to perform Gene Ontology (GO) and Kyoto Encyclopedia of Genes and Genomes (KEGG) analysis to explore biological processes and signaling pathways in Gusuibu for osteoporosis (Kanehisa et al., 2008; Yu et al., 2012; Consortium, 2015). An adjusted *p*-value of less than 0.05 was used to determine the enrichment term.

### Implementation of molecular docking

The Sankey diagram (http://sankeymatic.com/) reveals the correspondence between herbs, components, and targets by constructing the interrelationship between the active compounds of Gusuibu and the top 10 hub genes. AutoDock Vina was used to perform molecular docking between the top 10 hub genes and key active ingredients to predict their binding affinity (Trott and Olson, 2010). The PDB format of the target proteins and the MOL2 format of active compounds could be obtained from the RCSB protein data (http://www.rcsb.org/) and the PubChem database (https://pubchem.ncbi.nlm.nih.gov/) (Kanis, 2002). The smaller the molecular docking score, the more stable the binding affinity between target proteins and active compounds (Chang et al., 2020).

### Cell culture

C2C12 mesenchymal stem cells (ATCC) were cultured in DMEM with 10% fetal bovine serum (FBS, Gibco), 100U/mL penicillin G sodium and 100 μg/ml streptomycin sulfate in a humidified atmosphere at 37°C. Cells were plated in 6-well plates, cultured to 60–80% confluence and treated with kaempferol.

### Reverse transcription-quantitative PCR

Kaempferol (1,000 ng/ml; Sigma) was used to stimulate C2C12 cells for 24 h before harvest. TRIzol (Invitrogen Life Technologies, Carlsbad, CA, United States) was used to extract total RNA from cultured cells as previously described (Tang et al., 2012). RNA purity and integrity were determined by the RNA 6000 Nano assay with an Agilent Bioanalyzer 2,100 (Agilent Technologies, Santa Clara, CA). 1 μg total RNA was reverse transcribed into cDNA using the GoScript Reverse Transcription System (Promega Corp., Madison, WI, United States) based on the manufacturer protocol. qPCR was performed in triplicate using SYBR-Green SuperReal PreMix Plus [Tiangen Biotech (Beijing) Co., Ltd., China] and the iQ5 PCR system (Bio-Rad Laboratories, Inc., Hercules, CA, United States). The reaction conditions were as follows: 95°C for 30 s, and 40 cycles of 95°C for 10 s and 60°C for 30 s. Data were reported as cycle threshold (Ct) values. The 2^−ΔΔ^Ct method was used to compare the RNA expressions. RNA levels were normalized to glyceraldehyde-3-phosphate dehydrogenase (GAPDH) levels.

### Statistical analysis

All qPCR experiments were performed in triplicate. Data were expressed as the mean ± SD. Comparisons were made between groups by Student’s t test with *p* < 0.05 being considered as statistically significant.

## Results

### Pharmacodynamic compounds and potential target genes of gusuibu

A total of 131 compounds in Gusuibu (*Rhizoma Drynariae*) were obtained through the TCMSP database (Number: 71), TCMID database (Number: 53), and BATMAN-TCM platform (Number: 7). Based on the respective standards of TCMSP database (OB ≥ 30% and DL ≥ 0.18) and BATMAN-TCM platform (Score cutoff>20, adjusted *p*-value < 0.05) along with the existence of target proteins, a total of 24 active compounds of Gusuibu were screened. The corresponding target proteins were deduplicated, and a total of 331 target proteins were obtained. The UniProt database was used to convert the target proteins predicted by Gusuibu’s bioactive compounds into gene names. Table 1 includes the basic information of active compounds in Gusuibu.

**TABLE 1:** Basic information of active compounds in Gusuibu.

Molecule ID	Molecule name	OB (%)	DL	2D structure	InChI key	PubChem CID
MOL000006	Luteolin	36.16	0.25	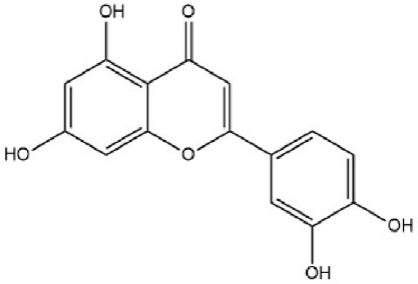	IQPNAANSBPBGFQ-UHFFFAOYSA-N	5280445
MOL000422	Kaempferol	41.88	0.24	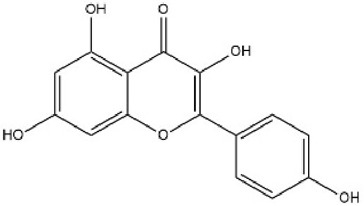	IYRMWMYZSQPJKC-UHFFFAOYSA-N	5280863
MOL004328	Naringenin	59.29	0.21	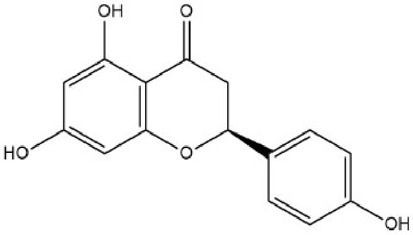	FTVWIRXFELQLPI-ZDUSSCGKSA-N	439246
MOL000358	Beta-sitosterol	36.91	0.75	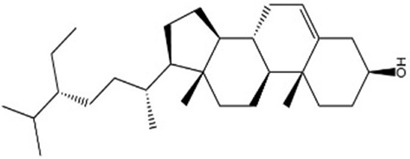	KZJWDPNRJALLNS-VJSFXXLFSA-N	222284
MOL000449	Stigmasterol	43.83	0.76	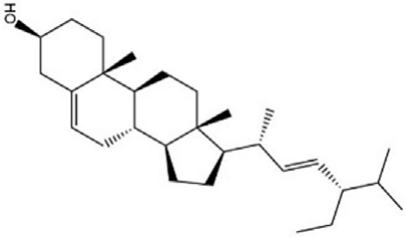	HCXVJBMSMIARIN-PHZDYDNGSA-N	5280794
MOL000492	(+)-catechin	54.83	0.24	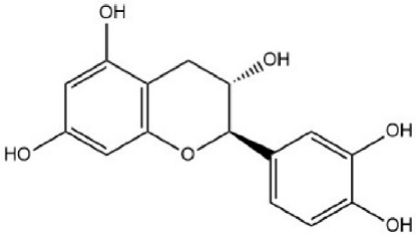	PFTAWBLQPZVEMU-DZGCQCFKSA-N	9064
MOL000569	Digallate	61.85	0.26	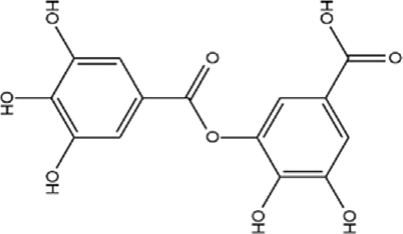	COVFEVWNJUOYRL-UHFFFAOYSA-N	341
MOL001040	(2R)-5,7-dihydroxy-2-(4-hydroxyphenyl)chroman-4-one	42.36	0.21	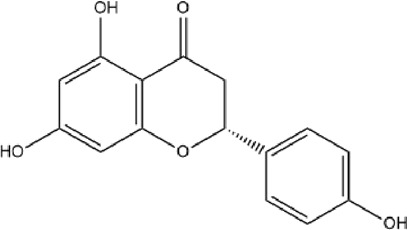	FTVWIRXFELQLPI-CYBMUJFWSA-N	667495
MOL001978	Aureusidin	53.42	0.24	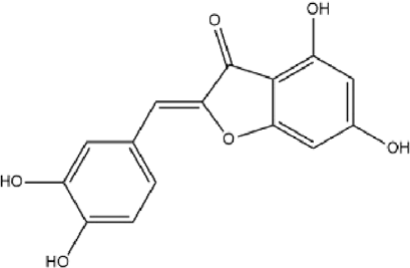	WBEFUVAYFSOUEA-PQMHYQBVSA-N	5281220
MOL002914	Eriodyctiol (flavanone)	41.35	0.24	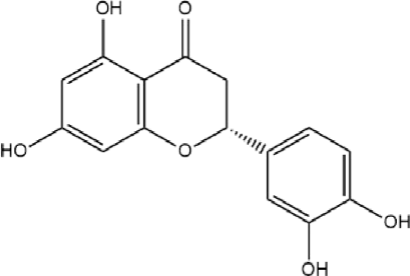	SBHXYTNGIZCORC-CYBMUJFWSA-N	373261
MOL005190	Eriodictyol	71.79	0.24	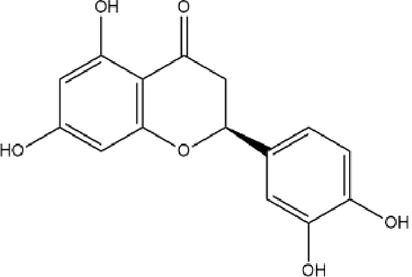	SBHXYTNGIZCORC-ZDUSSCGKSA-N	440735
MOL009061	22-Stigmasten-3-one	39.25	0.76	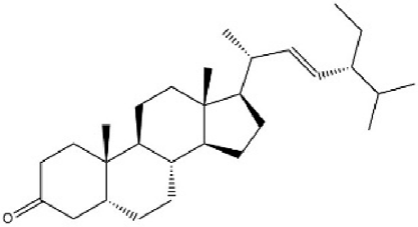	RTLUSWHIKFIQFU-ZBWVUXHASA-N	91692436
MOL009075	Cycloartenone	40.57	0.79	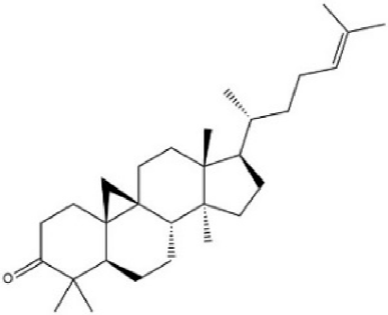	NAJCQAAOHKVCES-ZZOQNIIDSA-N	12305360
MOL009078	DavalliosideA_qt	62.65	0.51	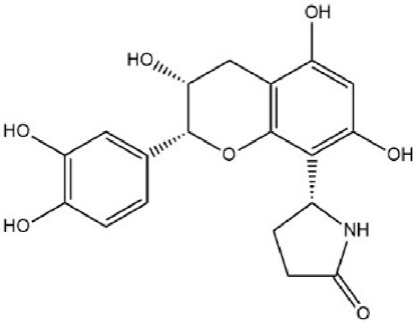	ZGIKMQJUZZSYOY-QEIWDELWSA-N	NR
MOL009091	Xanthogalenol	41.08	0.32	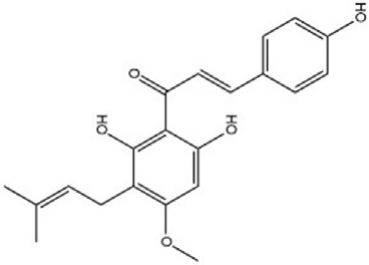	ALGFNVZQNNGHPA-YRNVUSSQSA-N	14309735
	Chitranone			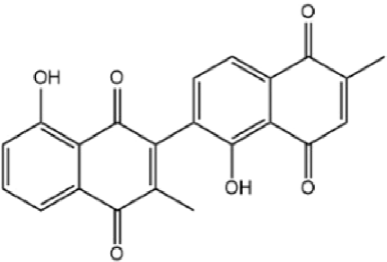	ITGPISXKMZIRAV-UHFFFAOYSA-N	633072
	Dihydrocaffeic acid			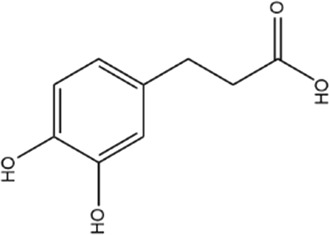	DZAUWHJDUNRCTF-UHFFFAOYSA-N	348154
	Kurarinone			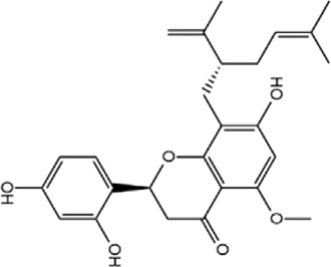	LTTQKYMNTNISSZ-MWTRTKDXSA-N	11982640
	Catechol			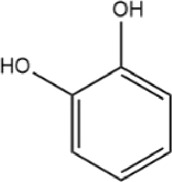	YCIMNLLNPGFGHC-UHFFFAOYSA-N	289
	Naringin			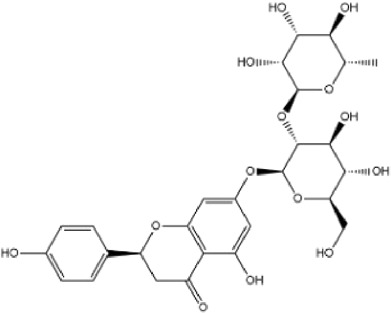	DFPMSGMNTNDNHN-ZPHOTFPESA-N	442428
	Campesterol			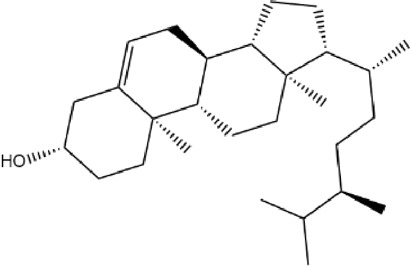	SGNBVLSWZMBQTH-PODYLUTMSA-N	173183
	Gamma-Sitosterol			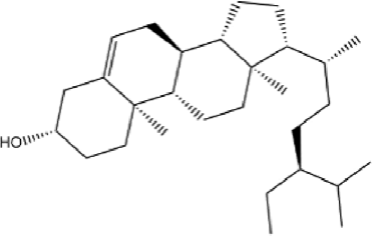	KZJWDPNRJALLNS-FBZNIEFRSA-N	457801
	Narirutin			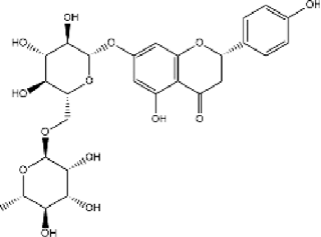	HXTFHSYLYXVTHC-AJHDJQPGSA-N	442431
	Hesperidin			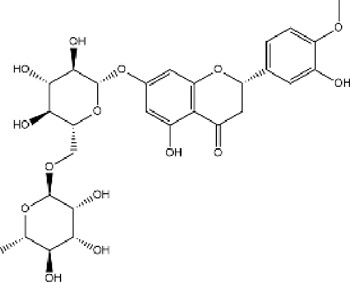	QUQPHWDTPGMPEX-QJBIFVCTSA-N	10621

Abbreviations: OB, Oral bioavailability; DL, Drug-likeness; NR, not reported.

### Potential therapeutic targets of gusuibu in the treatment of osteoporosis

**FIGURE 1:**
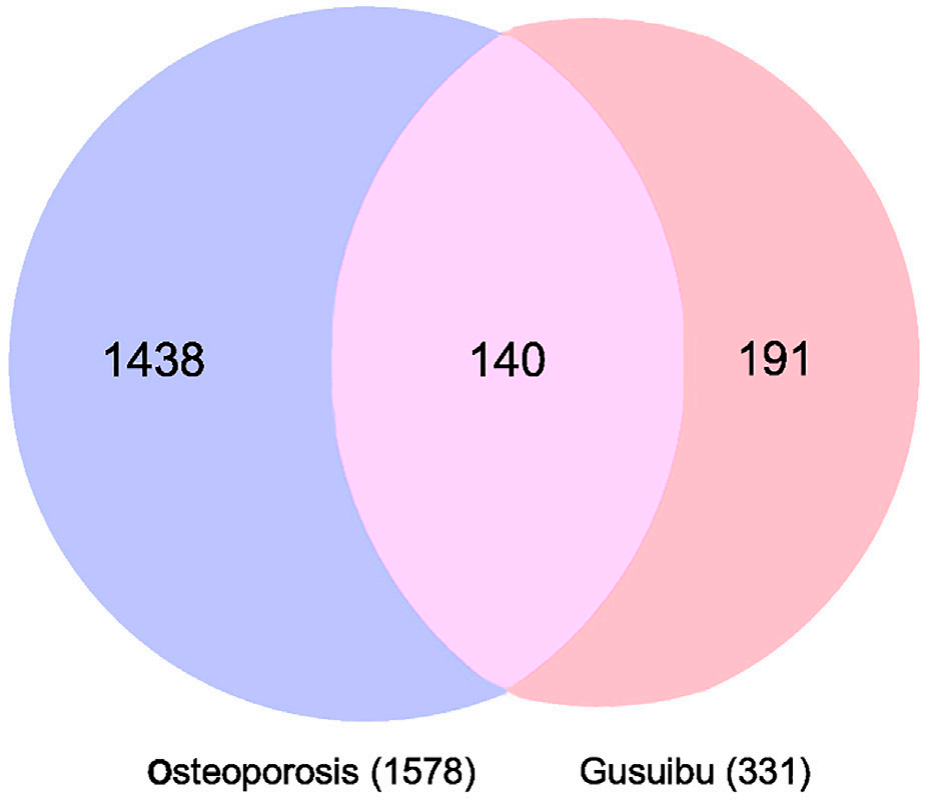
Venn diagram for the integrated analysis of the related targets of Gusuibu and osteoporosis.

**FIGURE 2:**
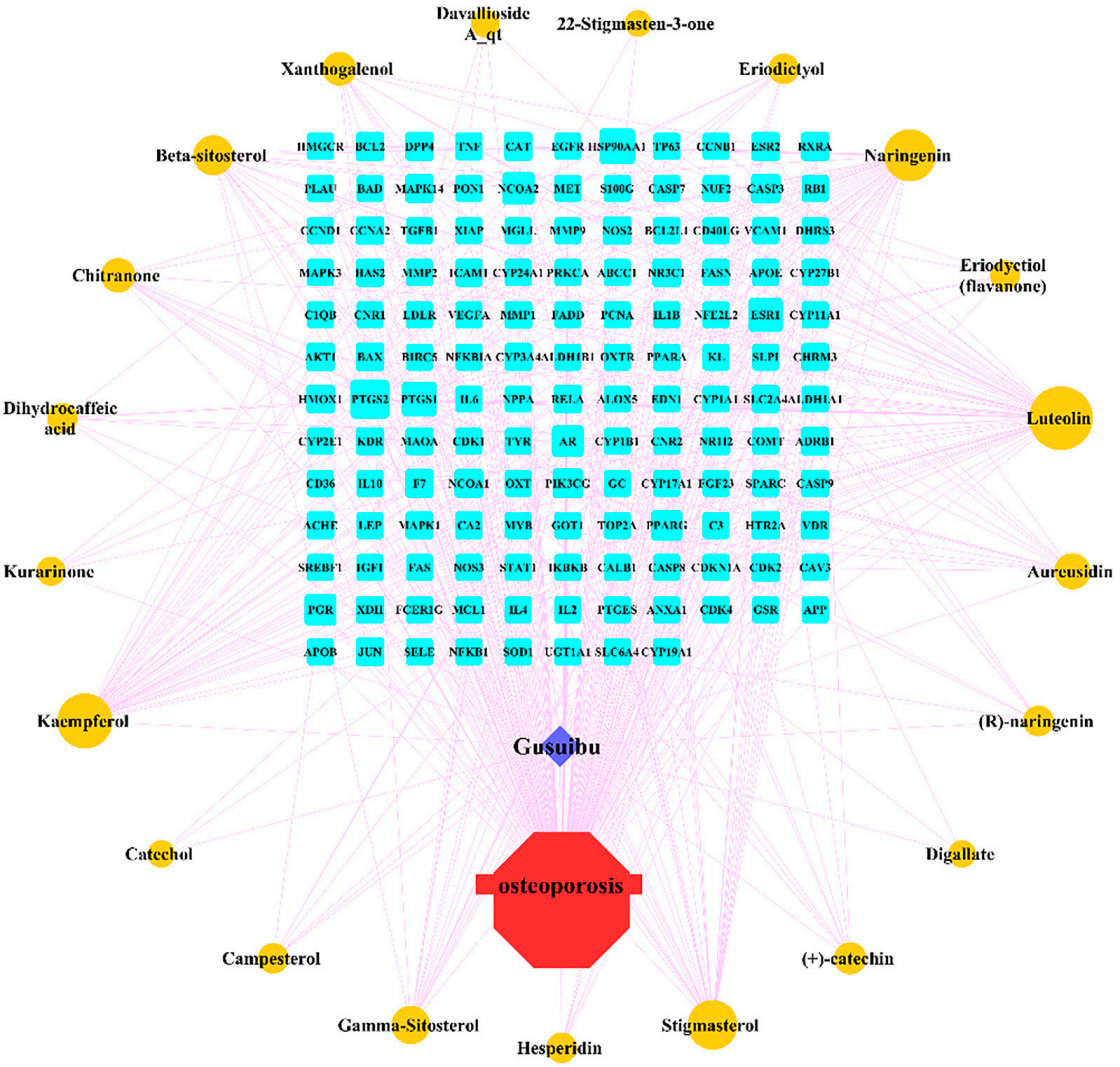
“Gusuibu-active compounds-target genes-osteoporosis” network. The red octagon represents osteoporosis; the blue diamond represents Gusuibu; the cyan rectangle represents the potential target; the brown ellipse represents the active compound contained in Gusuibu. The line between two nodes indicates that there is a relationship, and the size of each node indicates the number of relationships.

By searching the GeneCard, TTD, MalaCard, CTD, DisGeNET, and OMIM databases, a total of 1,578 potential therapeutic targets for osteoporosis were obtained. By constructing the Venn diagram of the targets regulated by Gusuibu’s active ingredients and the potential targets of osteoporosis, a total of 140 intersection targets were obtained, which are potential therapeutic targets of Gusuibu against osteoporosis as shown in Figure 1. Cytoscape software was used to construct the “Gusuibu-active compounds-target genes-osteoporosis” network for Gusuibu against osteoporosis as shown in Figure 2. There are three compounds (Cycloartenone, Narirutin, Naringin) corresponding to targets that are not intersecting genes, so there are 21 active compounds in the network.

### PPI network construction and exploration of potential hub genes for gusuibu against osteoporosis

**FIGURE 3:**
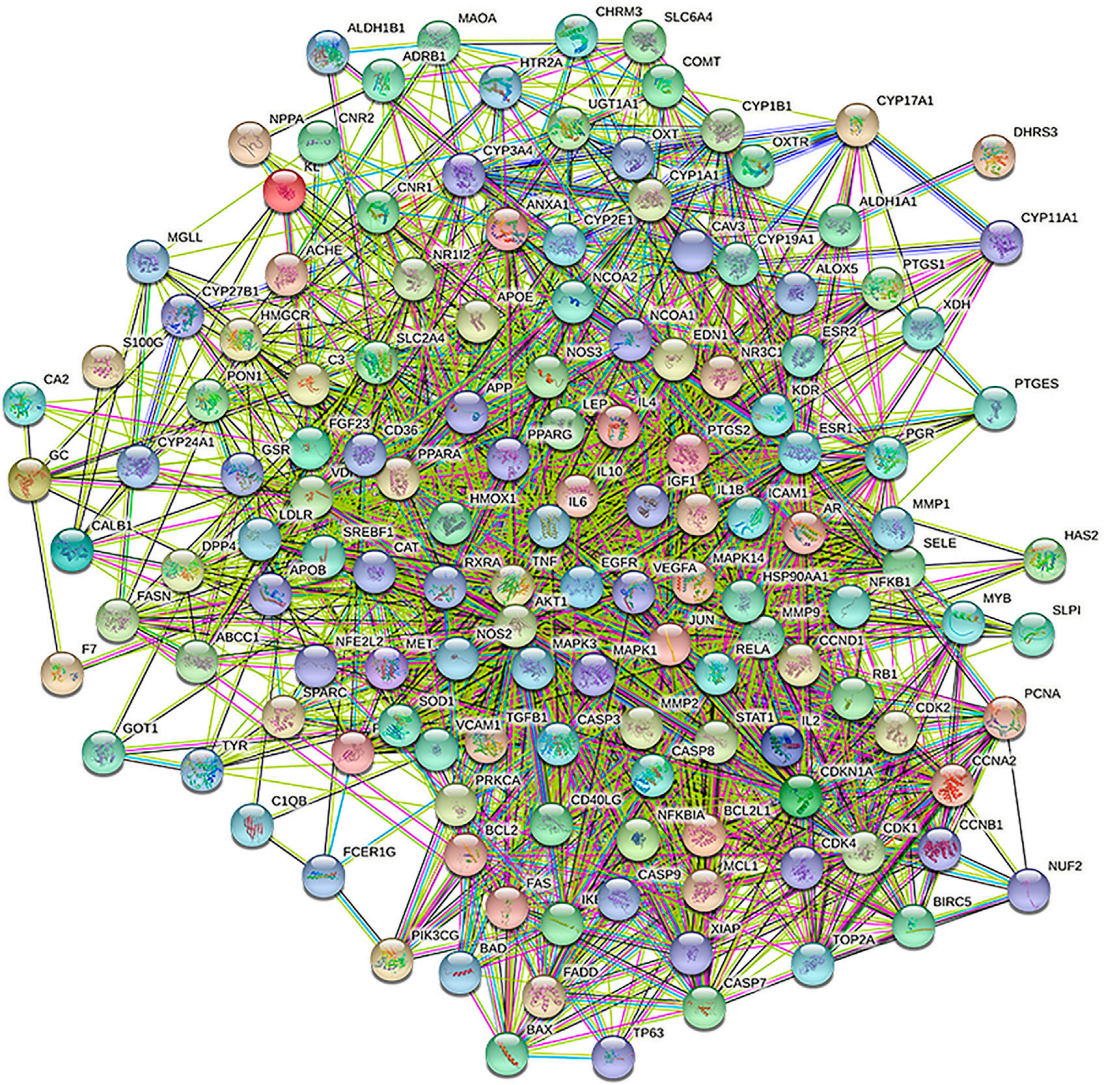
PPI network based on STRING database.

**FIGURE 4:**
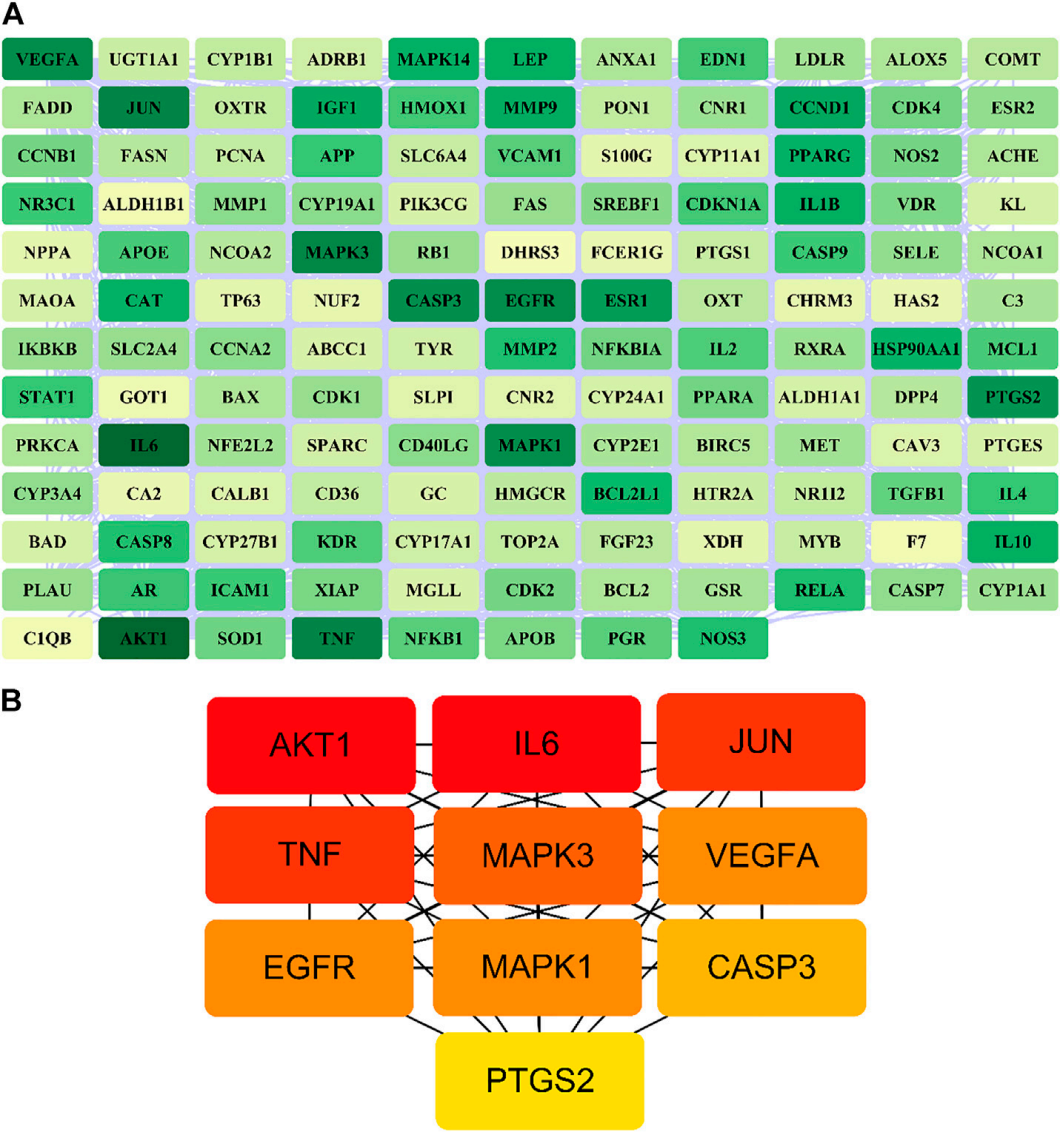
PPI network of potential target genes and top 10 hub genes for Gusuibu against osteoporosis. **(A)** PPI network constructed by using Cytoscape software. **(B)** The top 10 hub genes was identified by the Degree algorithm.

**FIGURE 5:**
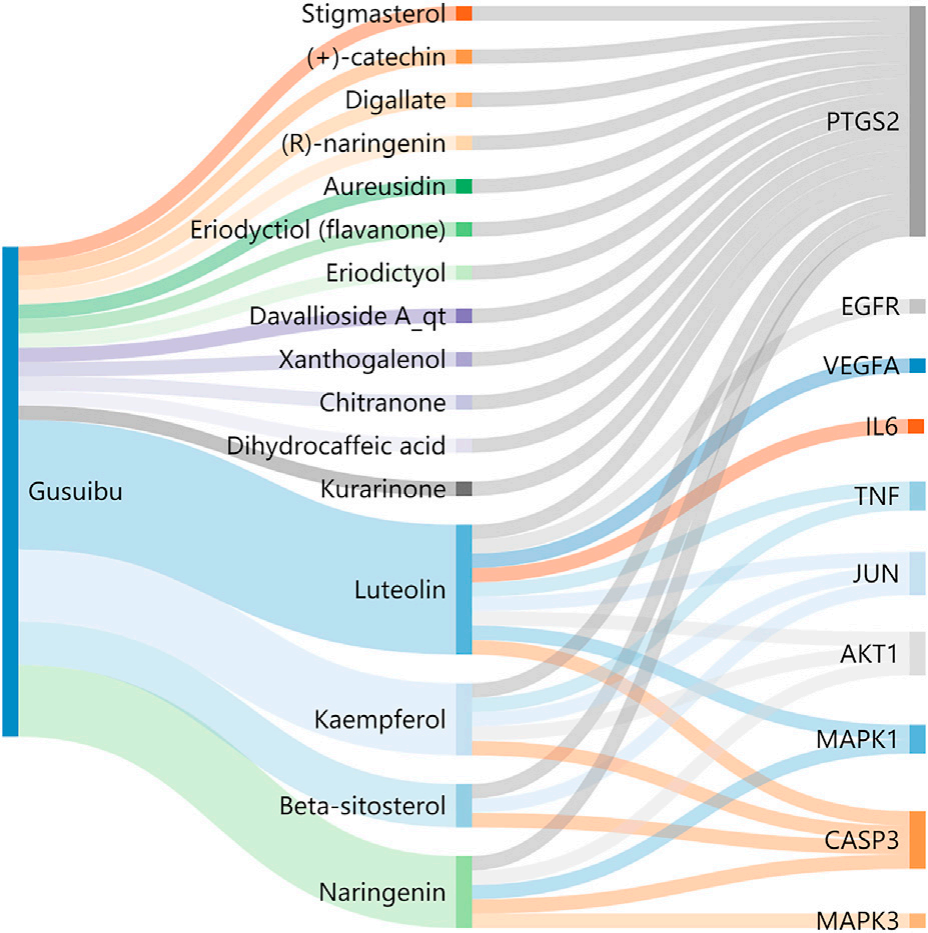
Sankey diagram. The blue vertical bar on the left represents Gusuibu, the middle bars represent the active ingredients of Gusuibu, and the right bars represent the top 10 hub genes. The size of the bars represents the number of correlations.

A total of 140 potential target genes of Gusuibu for treating osteoporosis were entered into the STRING database to obtain a PPI network, which involved 140 nodes and 2,199 edges (Figure 3). The data obtained was imported into Cytoscape software (version 3.7.2) for further visualization (Figure 4A). According to the 12 CytoHubba algorithms of Cytoscape software, the top 10 hub genes of Gusuibu for the treatment of osteoporosis were screened. A total of 37 different genes and the number of algorithms to which these genes belong were sorted in Table 2. The top 10 hub genes were consistent with the results of the Degree algorithm (Figure 4B). The Sankey diagram was constructed by using the top 10 ranked hub genes (AKT1, IL6, JUN, TNF, MAPK3, VEGFA, EGFR, MAPK1, CASP3, PTGS2) and the corresponding active compounds of Gusuibu. Luteolin targeted most of the hub genes while PTGS2 targeted most of the active compounds (Figure 5).

**TABLE 2:** Basic information of top 10 hub genes by 12 CytoHubba algorithms.

UniProt ID	Gene symbol	Protein names	Algorithms
P31749	AKT1	RAC-alpha serine/threonine-protein kinase	A, B, C, D, E, G, H, J, K, L
P05231	IL6	Interleukin-6	A, B, C, D, E, G, J, K, L
P05412	JUN	Transcription factor AP-1	A, B, C, D, G, H, J, K, L
P01375	TNF	Tumor necrosis factor	A, B, C, D, E, G, J, K, L
P42574	CASP3	Caspase-3	A, B, D, E, G, J, K, L
P28482	MAPK1	Mitogen-activated protein kinase 1	A, B, C, D, G, J, K, L
P27361	MAPK3	Mitogen-activated protein kinase 3	A, B, C, D, G, J, K, L
P35354	PTGS2	Prostaglandin G/H synthase 2	A, B, C, D, E, G, J, K
P15692	VEGFA	Vascular endothelial growth factor A	A, B, D, E, G, J, K
P00533	EGFR	Epidermal growth factor receptor	A, C, D, J, K, L
P03372	ESR1	Estrogen receptor	C, E, G, L
P14780	MMP9	Matrix metalloproteinase-9	B, E, H
P10275	AR	Androgen receptor	E, H
P04040	CAT	Catalase	C, L
P29965	CD40LG	CD40 ligand	I, H
P03956	MMP1	Interstitial collagenase	I, F
P16581	SELE	E-selectin	I, F
P98170	XIAP	E3 ubiquitin-protein ligase XIAP	I, H
P09917	ALOX5	Polyunsaturated fatty acid 5-lipoxygenase	H
Q92934	BAD	Bcl2-associated agonist of cell death	F
P55210	CASP7	Caspase-7	H
Q14790	CASP8	Caspase-8	I
P20309	CHRM3	Muscarinic acetylcholine receptor M3	F
P05108	CYP11A1	Cholesterol side-chain cleavage enzyme, mitochondrial	H
Q92819	HAS2	Hyaluronan synthase 2	F
P05362	ICAM1	Intercellular adhesion molecule 1	I
P22301	IL10	Interleukin-10	E
P60568	IL2	Interleukin-2	I
Q07820	MCL1	Induced myeloid leukemia cell differentiation protein Mcl-1	I
P10242	MYB	Transcriptional activator Myb	F
P25963	NFKBIA	NF-kappa-B inhibitor alpha	I
Q9BZD4	NUF2	Kinetochore protein Nuf2	F
P00749	PLAU	Urokinase-type plasminogen activator	H
O14684	PTGES	Prostaglandin E synthase	F
P03973	SLPI	Antileukoproteinase	F
P09486	SPARC	SPARC	F
P01137	TGFB1	Transforming growth factor beta-1 proprotein	I

A: Degree, B: Maximal Clique Centrality (MCC), C: Betweenness, D: closeness, E:BottleNeck, F: ClusteringCoefficient, G: Edge Percolated Component (EPC), H: EcCentricity, I: Density of Maximum Neighborhood Component (DMNC), J: Maximum Neighborhood Component (MNC), K: radiality, L: stress.

### Enrichment analysis of GO and KEGG pathway

**FIGURE 6:**
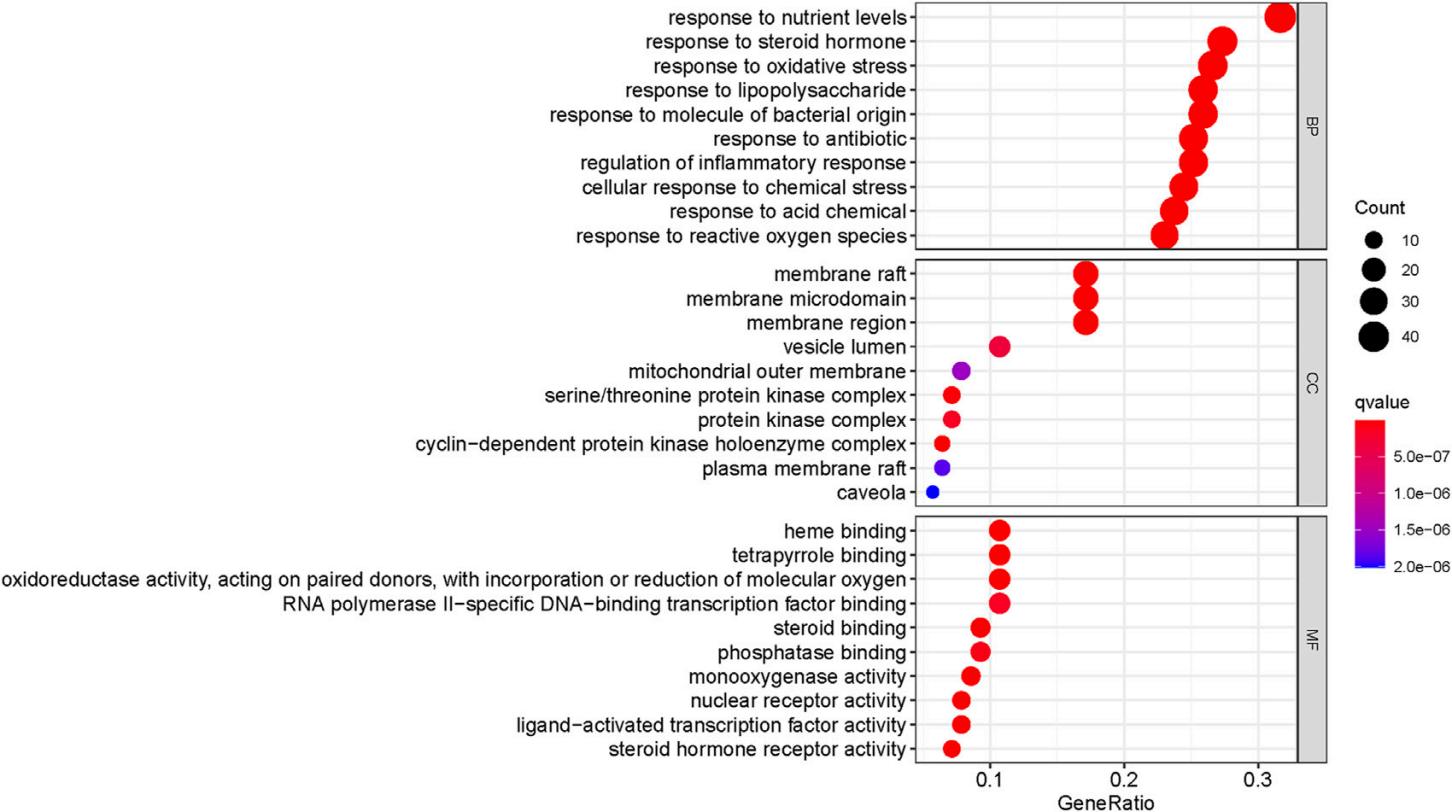
Bubble chart of the top 10 GO enriched items.

The Cluster Profiler package in R was used to perform GO enrichment analysis on 140 potential targets of Gusuibu in the treatment of osteoporosis. 2601 GO items (adjusted, *p* < 0.05) were obtained, including 2,398 biological process (BP) items, 75 cellular component (CC) terms and 128 molecular function (MF) items. As shown in Figure 6, the top 10 enrichment results of GO-BP, GO-CC, and GO-MF were visualized by bubble chart.

**FIGURE 7:**
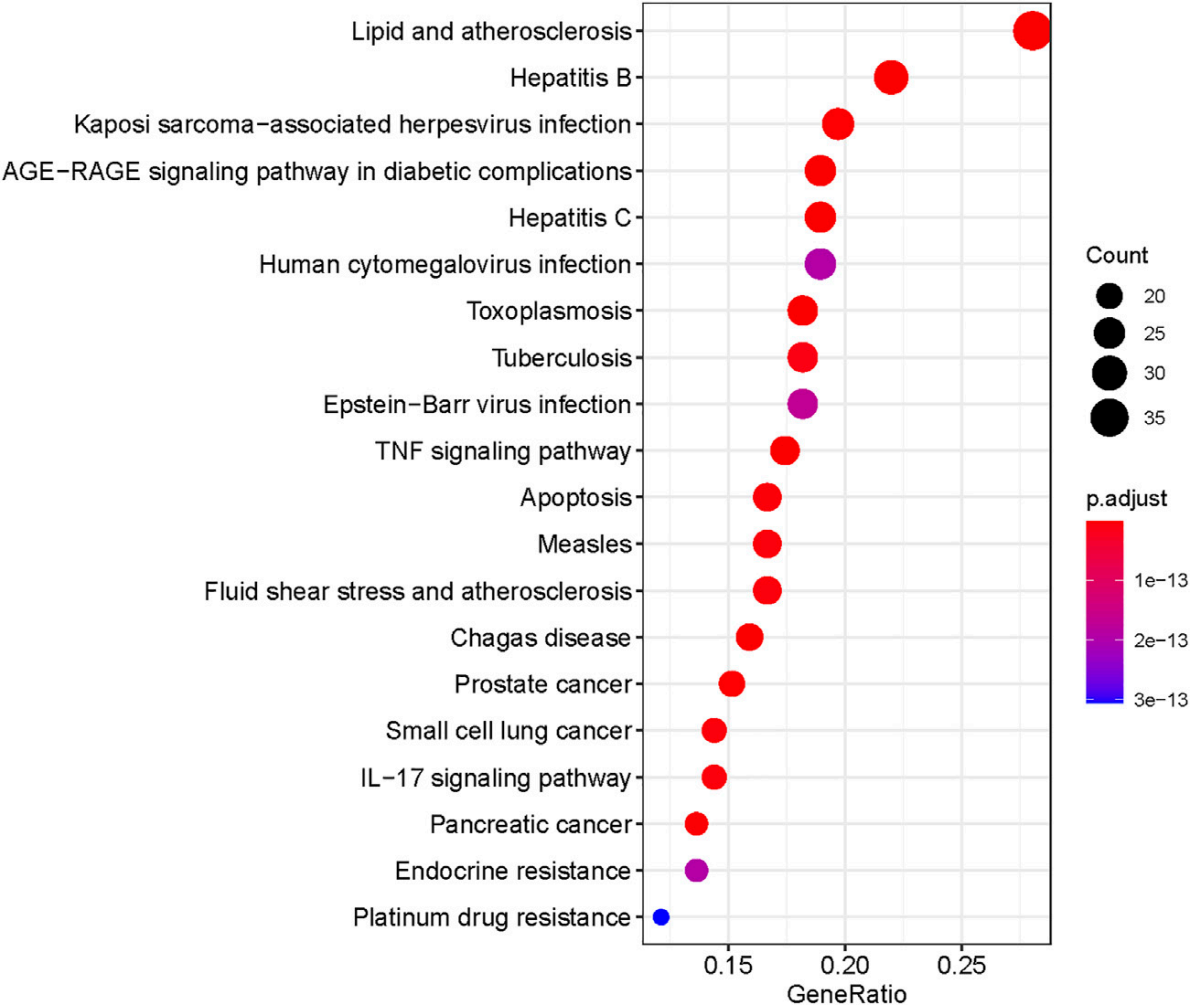
Bubble chart of the top 20 KEGG pathway enriched items.

A total of 166 KEGG enriched pathways (adjusted, *p* < 0.05) were obtained through the above R package. As shown in Figure 7, the top 20 KEGG pathway enrichment terms were visualized by bubble chart.

**FIGURE 8:**
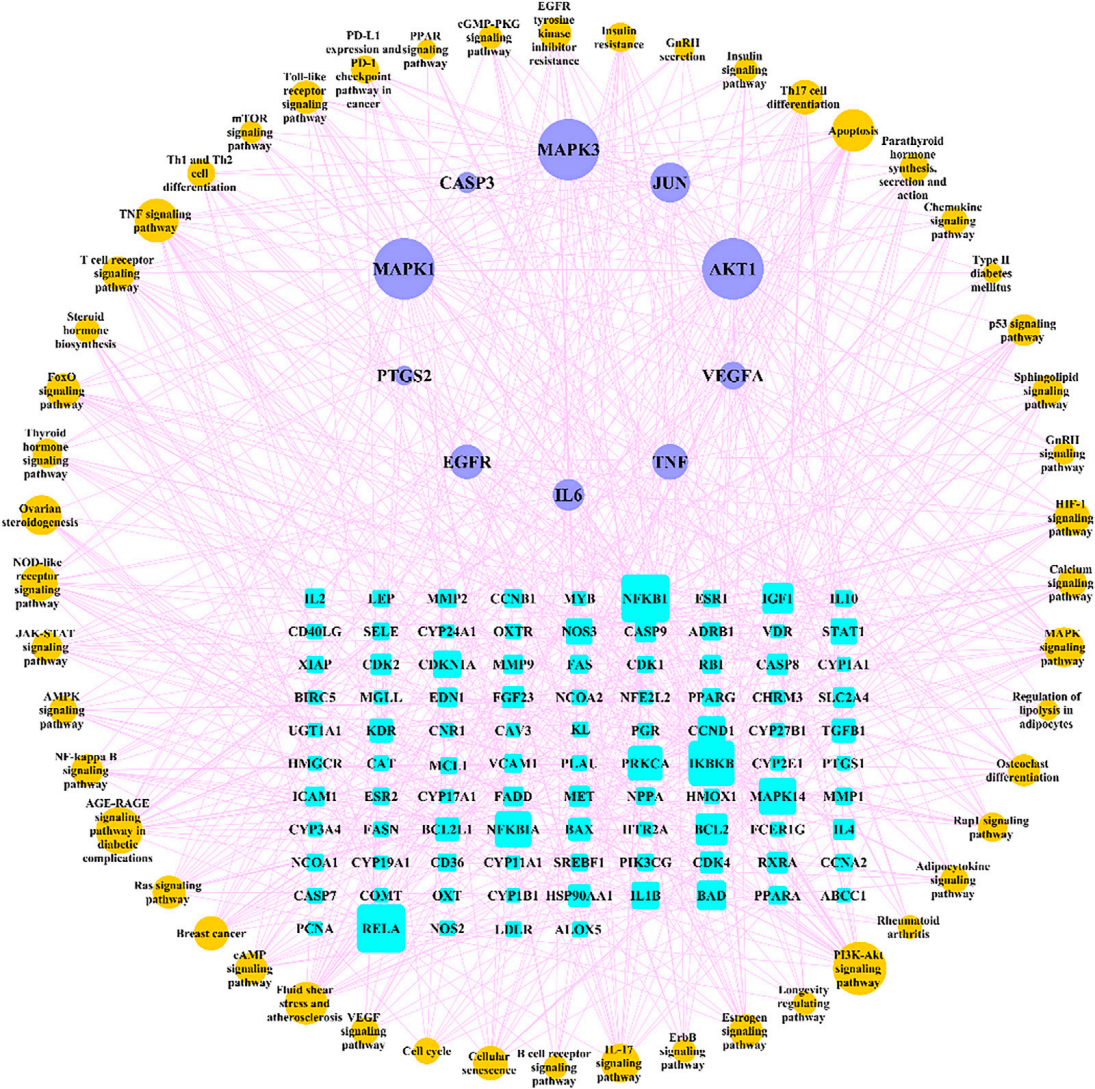
“Target genes-pathways” network. The cyan rectangle represents the potential target genes of Gusuibu against osteoporosis; the light blue ellipse represents the top 10 hub genes; the brown ellipse represents potential osteoporosis-related pathways.

We searched for osteoporosis in the KEGG database, and compared the retrieved relevant pathways with the enriched 166 pathways. A total of 50 pathways that may be related to osteoporosis were obtained, and we used Cytoscape software to construct a network diagram of potential target genes and pathways as shown in Figure 8.

### Molecular docking

**FIGURE 9:**
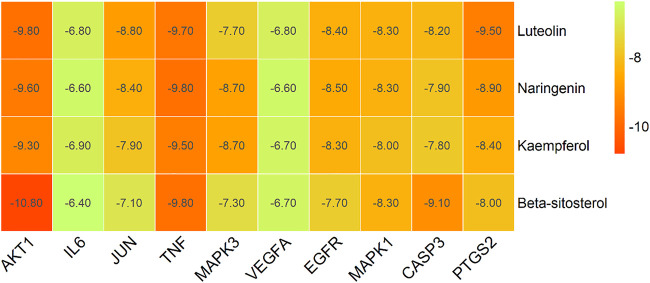
Heatmap of molecular docking scores.

Based on the Sankey diagram results, molecular docking was performed between the top 10 target proteins (AKT1, IL6, JUN, TNF, MAPK3, VEGFA, EGFR, MAPK1, CASP3, PTGS2) and four key active compounds (Luteolin, Naringenin, Kaempferol, and Beta-sitosterol) using AutoDock Vina. As shown in Figure 9, a heatmap was used to visualize the strongest affinity docking scores of top 10 target proteins and 4 key active compounds. The binding energy between the top 10 target proteins and the active compound is about −6.4 to −10.8 kcal mol^−1^, indicating that the key active compounds in Gusuibu bind well to the top 10 target proteins.

### Kaempferol induced the expression of osteoblast genes

**FIGURE 10:**
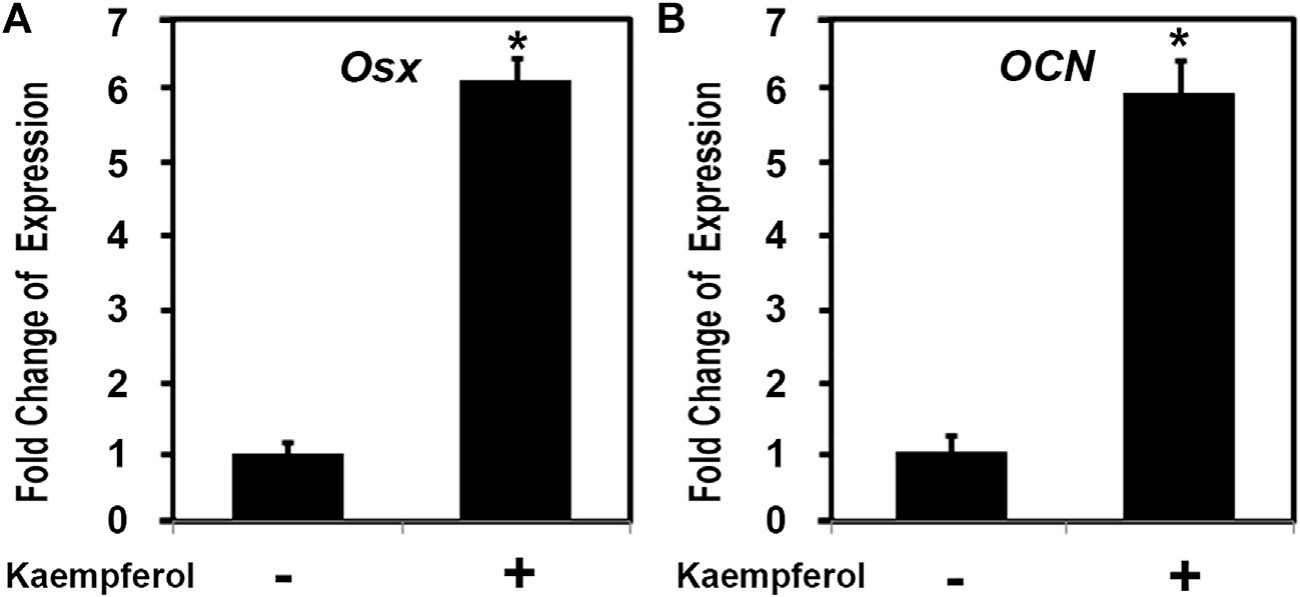
Kaempferol induced the expression of osteoblast genes. C2C12 cells were treated with 1,000 ng/ml kaempferol for 24 h. Total RNA was isolated and measured by real time RT-PCR. The RNA level from the control group was normalized to a value of 1. Values were presented as the mean ± S.D. *: A star indicates statistical significance compared to control group with *p* < 0.05. **(A)** Effect of kaempferol on OSX expression; **(B)** Effect of kaempferol on OCN expression.

To explore the possible role of one key active compound kaempferol in osteoblast genes, we examined the effect of kaempferol on osteoblast differentiation related gene expressions. C2C12 cells were cultured and treated as indicated with kaempferol (1,000 ng/ml, Sigma) for 24 h. Total RNA was isolated and measured by real time RT-PCR. As shown in Figure 10, after kaempferol stimulation osteoblast specific transcription factor Osterix (OSX) expression was upregulated by about 6.1 folds, while the expression of OSX downstream osteoblastic gene osteocalcin (OCN) increased by 5.9 fold. These observations demonstrated that kaempferol induced OSX gene expression.

**FIGURE 11:**
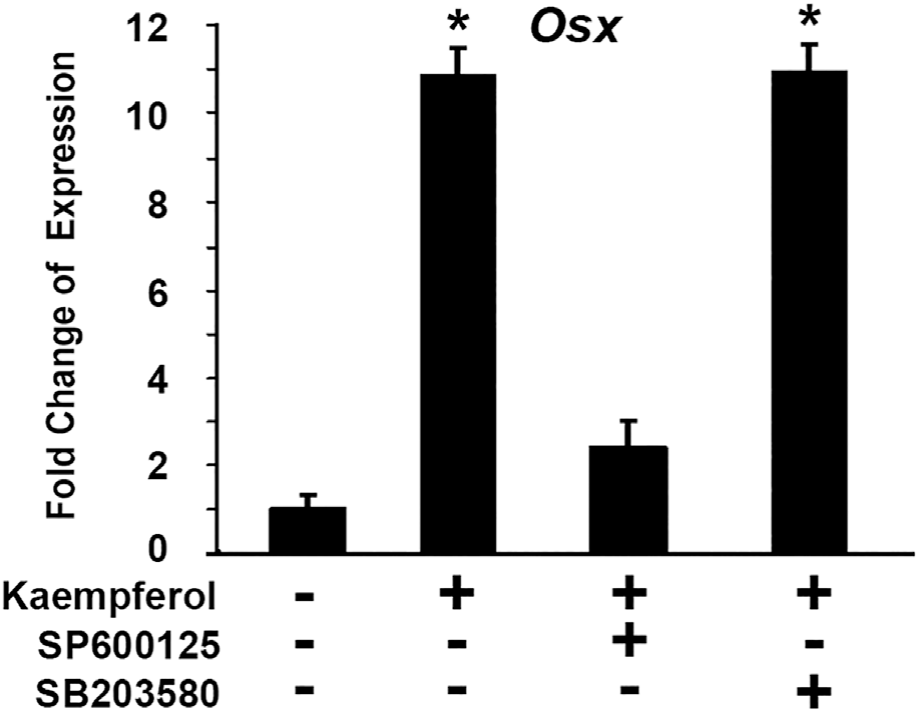
Kaempferol-induced OSX activation is mediated through JNK kinase pathway. Gene expressions were determined *via* qRT-PCR with or without kaempferol stimulation. The RNA level from the control group was normalized to a value of 1. *: A star indicates statistical significance compared to control group with *p* < 0.05. Specific inhibitors (50 μM SP600125 or 10 μM SB203580) were added as indicated.

We asked which pathway was involved in kaempferol induced OSX gene expression. To explore molecular mechanisms of kaempferol effect on OSX expression, we used a loss-of-function approach to examine possible pathways involved. The selective inhibitors were used: SB203580 as a specific inhibitor for p38 MAPK pathway, and SP600125 is a specific inhibitor for JNK kinase pathway. C2C12 cells were treated with kaempferol. Different inhibitors were added in the culture medium. As shown in Figure 11, kaempferol treatment led to OSX expression increase by 10.8-fold. Addition of 50 μM SP600125 almost abolished the increment of OSX expression induced by kaempferol. Kaempferol-induced OSX activation remained unchanged after treatment with 10 μM SB203580. The data indicate that kaempferol-induced OSX activation is mediated through JNK kinase pathway.

## Discussion

Osteoporosis has become an important health issue with aging (Zheng et al., 2020). For example, postmenopausal osteoporosis as a very common form of primary osteoporosis affects most postmenopausal women (Jackson and Mysiw, 2014). Due to severe side effects of current clinical osteoporosis drugs, more and more attention has been paid to possible drugs for the prevention and treatment of osteoporosis from natural products (Li et al., 2020a). Qianggu Capsule is a proprietary Traditional Chinese Medicine approved by CFDA to treat osteoporosis, and its active ingredient is total flavonoids of *Rhizoma Drynariae* (Gusuibu) (CSoOaBM, 2019). Studies have shown that Qianggu Capsule may prevent and treat osteoporosis by improving bone density and reducing bone loss (Wei et al., 2017). The raw material of Qianggu Capsules is the flavonoids of the herbal Gusuibu, but the mixture is not a single compound at all, and the underlying mechanism is not clear yet. Based on the network pharmacological approaches such as databases mining, PPI network construction, GO and KEGG enrichment analysis and molecular docking validation, this study aims to analyze the active ingredients and potential targets of Gusuibu for osteoporosis treatment and to explore possible mechanisms of Qianggu Capsule active ingredients.

Based on the principle of ADME (setting OB ≥ 30 and DL ≥ 0.18) (Tsaioun et al., 2016), we searched three herbal databases, including TCMSP, TCMID and BATMAN-TCM platform, and screened 24 active compounds from Gusuibu (131 initially, after removing duplicates and null targets), and finally 331 corresponding target proteins were found. The protein name of the target protein obtained was converted into a gene ID through the UniProt database. Subsequently, we searched 6 disease-related databases (GeneCards, MalaCards, DisGeNET, TTD, CTD, and OMIM) and obtained a total of 1,578 potential therapeutic targets for osteoporosis. After constructing Venn diagrams with potential targets of Gusuibu, we obtained 140 potential target genes of Gusuibu for osteoporosis treatment. “Gusuibu-active compounds-target genes-osteoporosis” network was established, which involved 163 nodes and 432 interactions. The PPI network of potential therapeutic targets had also been constructed, involving 140 nodes and 2,199 edges. Based on the CytoHubba 12 algorithms of Cytoscape software (Degree, Clustering Coefficient, DMNC, Bottle Neck, MCC, MNC, Radiality, EPC, EcCentricity, Closeness, Betweenness, Stress), the top 10 hub genes were obtained, including AKT1, IL6, JUN, TNF, MAPK3, VEGFA, EGFR, MAPK1, CASP3, PTGS2. The physiological processes regulated by the proteins encoded by these core target genes mainly include inflammatory response, cell proliferation and differentiation, apoptosis, migration, cell cycle progression, angiogenesis, immune response, endocrine metabolism, bone metabolism, reproductive function, growth and nutrition. Therefore, based on these results, it may be speculated that Gusuibu may play a role in resisting osteoporosis through the multiple processes.

Sankey diagram showed the one-to-one correspondence between the top 10 hub genes and the corresponding active compounds contained in Gusuibu [Luteolin, Naringenin, Kaempferol, Beta-sitosterol, (+)-catechin, (R)-naringenin, Aureusidin, Digallate, Eriodyctiol (flavanone), Eriodictyol, DavalliosideA_qt, Dihydrocaffeic acid, Kurarinone, Chitranone, Stigmasterol, Xanthogalenol]. Among them, Luteolin targeted nine hub genes, Naringenin targeted five hub genes, Kaempferol targeted five hub genes, Beta-sitosterol targeted three hub genes, and the others targeted one hub gene each. Luteolin is a flavonoid found in many herbal extracts with antioxidant, anti-inflammatory and anti-tumor activities, and has been shown to reduce glucocorticoid-induced osteoporosis by modulating the ERK/Lrp-5/GSK-3β signaling pathway *in vitro* and *in vivo* (Jing et al., 2019). We summarized the relevant literature exploring the possible mechanisms of Qianggu Capsule and its compounds for osteoporosis treatment (Liu et al., 2001; Wong et al., 2007; Wang et al., 2008; Chen et al., 2009; Hung et al., 2010; Guo et al., 2011; Huang et al., 2011; Ouyang et al., 2012; Li et al., 2017b; Wei et al., 2017; Zhang et al., 2017; Jing et al., 2019; Dong et al., 2020; Ruangsuriya et al., 2020; Shen et al., 2020; Liu et al., 2021) in Table 3.

**TABLE 3:** Studies related to the regulation of bone metabolism by Qianggu Capsule and its active compounds.

Chinese herbal medicine or compounds	Study	Organism	Possible pharmacological mechanisms	References
Qianggu Capsule (total flavonoids of *Rhizoma Drynariae*)	Wei et al. (2017)	Human	Improving BMD	Wei et al. (2017)
	Zhang et al. (2017)	Human	Improving BMD	Zhang et al. (2017)
	Li et al. (2017b)	Rat	Enhancing the expression of TGF-β1 and promoting bone metabolism	Li et al. (2017b)
	Ouyang et al. (2012)	Human	Improving BMD	Ouyang et al. (2012)
	Huang et al. (2011)	Rat	Promoting the proliferation and decrease the apoptosis of osteoblasts by improving the ratio of Bcl-2 mRNA to Bax mRNA	Huang et al. (2011)
	Chen et al. (2009)	Rat	Promoting bone metabolism	Chen et al. (2009)
Gusuibu (*RhizomaDrynariae*)	Shen et al. (2020)	Rat	Enhancing angiogenic-osteogenic coupling during distraction osteogenesis by promoting type H vessel formation through PDGF-BB/PDGFR-β instead of HIF-1α/VEGF axis	Shen et al. (2020)
	Hung et al. (2010)	Rat	Promoting osteoblast maturation by regulating bone differentiation-related gene expression	Hung et al. (2010)
	Wang et al. (2008)	Rat	Promoting bone healing	Wang et al. (2008)
	Wong et al. (2007)	Mouse	Promoting bone healing	Wong et al. (2007)
	Liu et al. (2001)	Rat	Promoting bone healing	Liu et al. (2001)
Luteolin	Jing et al. (2019)	Mouse	Luteolin reduced glucocorticoid-induced osteoporosis by modulating the ERK/Lrp-5/GSK-3β signaling pathway	Jing et al. (2019)
Naringin	Dong et al. (2020)	Rabbit	Naringin may be good natural BMP regulator in bone tissue engineering	Dong et al. (2020)
Naringin and Naringenin	Guo et al. (2011)	Mouse	Naringin and Naringenin revealed a double directional adjusting function of estrogenic and anti-estrogenic activities	Guo et al. (2011)
Kaempferol	Liu et al. (2021)	Rat	Kaempferol promotes BMSC osteogenic differentiation and improves osteoporosis by downregulating miR-10a-3p and upregulating CXCL12	Liu et al. (2021)
Beta-sitosterol	Ruangsuriya et al. (2020)	Human	Beta-sitosterol triggered the molecules involved in bone formation and the molecules inhibiting bone resorption	Ruangsuriya et al. (2020)

Abbreviations: BMD, bone mineral density; BMSC, bone marrow mesenchymal stem cell.

We further analyzed the potential therapeutic genes of Gusuibu against osteoporosis by GO and KEGG pathway enrichment analysis. We used osteoporosis as a search term in the KEGG database and obtained ten directly related pathways, including osteoclast differentiation (hsa04380), AGE-RAGE signaling pathway in diabetic complications (hsa04933), Endocrine and other factor-regulated calcium reabsorption (hsa04961), Mineral absorption (hsa04978), Wnt pathway (hsa04310), MAPK pathway (hsa04010), Apoptosis (hsa04210), Chemokine pathway (hsa04062), T cell receptor pathway (hsa04660), and B cell receptor pathway (hsa04662). Five of the top 10 hub genes (AKT1, TNF, MAPK3, JUN, MAPK1) are enriched in osteoclast differentiation. Eight of the top 10 hub genes (AKT1, IL6, CASP3, TNF, VEGFA, MAPK3, JUN, MAPK1) are enriched in AGE-RAGE pathway in diabetic complications. Eight of the top 10 hub genes (AKT1, CASP3, TNF, VEGFA, MAPK3, JUN, EGFR, MAPK1) are enriched in MAPK pathway. Six of the top 10 hub genes (AKT1, CASP3, TNF, MAPK3, JUN, MAPK1) are enriched in Apoptosis. Three of the top 10 hub genes (AKT1, MAPK3, MAPK1) are enriched in Chemokine pathway. Five of the top 10 hub genes (AKT1, TNF, MAPK3, JUN, MAPK1) are enriched in T cell receptor pathway. Four of the top 10 hub genes (AKT1, MAPK3, JUN, MAPK1) are enriched in B cell receptor pathway. The three pathways are not enriched including Endocrine and other factor-regulated calcium reabsorption, Mineral absorption, and Wnt pathway.

To search for potentially relevant pathways, we obtained 50 enriched pathways as osteoporosis-related pathways and used Cytoscape software to construct the “pathway-gene” network. Studies have shown that there are many pathways associated with osteoporosis, which are mainly related to bone metabolism, inflammatory response, immune response, endocrine metabolism and apoptosis (Liu et al., 2020). Based on these analyses, Gusuibu may exert anti-osteoporosis effects through many pathways and hub genes.

As an osteoblast-specific transcription factor OSX is required for bone formation and osteoblast differentiation (Nakashima et al., 2002; Zhang et al., 2008). OSX was originally discovered as a BMP2 inducible gene in C2C12 mesenchymal stem cells, and OSX knock-out mice lack bone formation (Nakashima et al., 2002). OCN is a well-known downstream osteoblastic gene of OSX. There are three critical transcription factors required for osteoblast differentiation and bone formation: IHH, RUNX2 and OSX. OSX is the only osteoblast-specific transcription factor identified so far, and it is only expressed in cells in the bone matrix and the inner (endosteum) and outer (periosteum) bone surfaces. OSX discovery as a master regulator of bone formation and osteoblast differentiation opens a new window to bone biology. As far as we know, this is the first study to discover that one active compound kaempferol in Gusuibu activated the expression of osteoblast specific transcription factor OSX through JNK kinase pathway.

In summary, through network pharmacology, we explored the potential mechanisms by which Gusuibu exerts its anti-osteoporosis effects with a multi-component, multi-target, and multi-pathway profile. Potential therapeutic targets for Gusuibu to exert anti-osteoporosis effects include AKT1, IL6, JUN, TNF, MAPK3, VEGFA, EGFR, MAPK1, CASP3, PTGS2. Most of the key active compounds in Gusuibu are flavonoids, mainly including Luteolin, Naringenin, Kaempferol, and Beta-sitosterol. Interestingly, kaempferol was identified as an upregulator of OSX.

## Data Availability

The original contributions presented in the study are included in the article/Supplementary Material, further inquiries can be directed to the corresponding author.

## References

[B1] Amberger J. Hamosh A. (2017). Searching online mendelian inheritance in man (OMIM): A knowledgebase of human genes and genetic phenotypes. Curr. Protoc. Bioinforma. 581, 1–2. 10.1002/cpbi.27 PMC5662200 28654725

[B2] Chang J. Liu L. Wang Y. Sui Y. Li H. Feng L. (2020). Investigating the multitarget mechanism of traditional Chinese medicine prescription for cancer-related pain by using network pharmacology and molecular docking approach. Evid. Based. Complement. Altern. Med. 2020, 7617261. 10.1155/2020/7617261 PMC7673937 33224254

[B3] Chen L. Chen L. Chen H. Guo X. Xu H. Zhang G. (2009). Skeletal biomechanical effectiveness of retinoic acid on induction of osteoporotic rats treated by alendronate and qianggu capsules. Zhonghua yi xue za zhi 89 (27), 1930–1933. 19953920

[B4] Chin C. Chen S. Wu H. Ho C. Ko M. Lin C. (2014). cytoHubba: identifying hub objects and sub-networks from complex interactome. BMC Syst. Biol. S11, S11. 10.1186/1752-0509-8-S4-S11 PMC4290687 25521941

[B5] Consortium T. G. O. (2015). Gene Ontology Consortium: Going forward. Nucleic Acids Res. 43, D1049–D1056. 10.1093/nar/gku1179 25428369 PMC4383973

[B6] CSoOaBM Research (2019). Guidelines for the diagnosis and management of primary osteoporosis (2017). Chin. J. Osteoporos. 25 (3), 281.

[B7] Davis A. Grondin C. Johnson R. Sciaky D. Wiegers J. Wiegers T. (2020). Comparative Toxicogenomics database (CTD): Update 2021. Nucleic acids Res. 49, D1138–D1143. 10.1093/nar/gkaa891 PMC7779006 33068428

[B8] Dong G. Ma T. Li C. Chi C. Su C. Huang C. (2020). A study of Drynaria fortunei in modulation of BMP–2 signalling by bone tissue engineering. Turk. J. Med. Sci. 50 (5), 1444–1453. 10.3906/sag-2001-148 32252500 PMC7491309

[B9] Ge J. Xie L. Chen J. Li S. Xu H. Lai Y. (2018). Liuwei dihuang Pill treats postmenopausal osteoporosis with shen (kidney) yin deficiency via janus kinase/signal transducer and activator of transcription signal pathway by up-regulating cardiotrophin-like cytokine factor 1 expression. Chin. J. Integr. Med. 24 (6), 415–422. 10.1007/s11655-016-2744-2 28028720

[B10] Guo D. Wang J. Wang X. Luo H. Zhang H. Cao D. (2011). Double directional adjusting estrogenic effect of naringin from Rhizoma drynariae (Gusuibu). J. Ethnopharmacol. 138 (2), 451–457. 10.1016/j.jep.2011.09.034 21964193

[B11] Huang L. Xie D. Yu Y. Liu H. Shi Y. Shi T. (2018). Tcmid 2.0: A comprehensive resource for TCM. Nucleic Acids Res. 46, D1117–D1120. 10.1093/nar/gkx1028 29106634 PMC5753259

[B12] Huang Z. Ouyang G. Xiao L. Li N. Gao H. He Y. (2011). Effects of Drynaria total flavonoids on apoptosis of osteoblasts mediated by tumor necrosis factor-α. Zhong xi yi jie he xue bao 9 (2), 173–178. 10.3736/jcim20110210 21288453

[B13] Hung T. Chen T. Liao M. Ho W. Liu D. Chuang W. (2010). Drynaria fortunei J. Sm. promotes osteoblast maturation by inducing differentiation-related gene expression and protecting against oxidative stress-induced apoptotic insults. J. Ethnopharmacol. 131 (1), 70–77. 10.1016/j.jep.2010.05.063 20554009

[B14] Jackson R. Mysiw W. (2014). Insights into the epidemiology of postmenopausal osteoporosis: The women's health initiative. Semin. Reprod. Med. 32 (6), 454–462. 10.1055/s-0034-1384629 25321423

[B15] Jing Z. Wang C. Yang Q. Wei X. Jin Y. Meng Q. (2019). Luteolin attenuates glucocorticoid-induced osteoporosis by regulating ERK/Lrp-5/GSK-3β signaling pathway *in vivo* and *in vitro* . J. Cell. Physiol. 234 (4), 4472–4490. 10.1002/jcp.27252 30192012

[B16] Kanehisa M. Araki M. Goto S. Hattori M. Hirakawa M. Itoh M. (2008). KEGG for linking genomes to life and the environment. Nucleic Acids Res. 36, D480–D484. 10.1093/nar/gkm882 18077471 PMC2238879

[B17] Kanis J. A. (2002). Diagnosis of osteoporosis and assessment of fracture risk. Lancet (London, Engl. 359–1936. 10.1016/S0140-6736(02)08761-5 12057569

[B18] Li G. Xu Q. Han K. Yan W. Huang C. (2020). Experimental evidence and network pharmacology-based analysis reveal the molecular mechanism of Tongxinluo capsule administered in coronary heart diseases. Biosci. Rep. 40 (10), BSR20201349. 10.1042/BSR20201349 32990315 PMC7560518

[B19] Li J. Jia Y. Chai L. Mu X. Ma S. Xu L. (2017). Effects of Chinese herbal formula erxian decoction for treating osteoporosis: A systematic review. Clin. Interv. Aging 12, 45–53. 10.2147/CIA.S117597 28115834 PMC5221555

[B20] Li J. Wang W. Feng G. Du J. Kang S. Li Z. (2020). Efficacy and safety of duhuo jisheng decoction for postmenopausal osteoporosis: A systematic review and meta-analysis. Evid. Based. Complement. Altern. Med. 2020, 6957825. 10.1155/2020/6957825 PMC7512089 33014108

[B21] Li M. Wang Y. Liao N. Li J. Dong Q. (2017). Changes of TGF-β1 expression during orthodontic tooth movement in rats with osteoporosis. Shanghai kou qiang yi xue 26 (1), 17–20. 28474060

[B22] Liu H. Chen R. Jian W. Lin Y. (2001). Cytotoxic and antioxidant effects of the water extract of the traditional Chinese herb gusuibu (Drynaria fortunei) on rat osteoblasts. J. Formos. Med. Assoc. = Taiwan yi zhi 100 (6), 383–388. 11480247

[B23] Liu H. Yi X. Tu S. Cheng C. Luo J. (2021). Kaempferol promotes BMSC osteogenic differentiation and improves osteoporosis by downregulating miR-10a-3p and upregulating CXCL12. Mol. Cell. Endocrinol. 520, 111074. 10.1016/j.mce.2020.111074 33157164

[B24] Liu W. Jiang Z. Chen Y. Xiao P. Wang Z. Huang T. (2020). Network pharmacology approach to elucidate possible action mechanisms of Sinomenii Caulis for treating osteoporosis. J. Ethnopharmacol. 257, 112871. 10.1016/j.jep.2020.112871 32325182

[B25] Liu Z. Guo F. Wang Y. Li C. Zhang X. Li H. (2016). BATMAN-TCM: A bioinformatics analysis tool for molecular mechANism of traditional Chinese medicine. Sci. Rep. 6, 21146. 10.1038/srep21146 26879404 PMC4754750

[B26] Nakashima K. Zhou X. Kunkel G. Zhang Z. Deng J. M. Behringer R. R. (2002). The novel zinc finger-containing transcription factor osterix is required for osteoblast differentiation and bone formation. Cell. 108, 17–29. 10.1016/s0092-8674(01)00622-5 11792318

[B27] Ouyang G. Feng X. Xiao L. Huang Z. Xia Q. Zhu F. (2012). Effects of Chinese herbal medicine qianggu capsule on patients with rheumatoid arthritis-induced osteoporosis: A report of 82 cases. Zhong Xi Yi Jie He Xue Bao 10 (12), 1394–1399. 10.3736/jcim20121210 23257132

[B28] Piñero J. Ramírez-Anguita J. Saüch-Pitarch J. Ronzano F. Centeno E. Sanz F. (2020). The DisGeNET knowledge platform for disease genomics: 2019 update. Nucleic Acids Res. 48, D845–D855. 10.1093/nar/gkz1021 31680165 PMC7145631

[B29] Rappaport N. Twik M. Plaschkes I. Nudel R. Iny Stein T. Levitt J. (2017). MalaCards: An amalgamated human disease compendium with diverse clinical and genetic annotation and structured search. Nucleic Acids Res. 45, D877–D887. 10.1093/nar/gkw1012 27899610 PMC5210521

[B30] Ru J. Li P. Wang J. Zhou W. Li B. Huang C. (2014). Tcmsp: A database of systems pharmacology for drug discovery from herbal medicines. J. Cheminform. 6 (13). 10.1186/1758-2946-6-13 PMC4001360 24735618

[B31] Ruangsuriya J. Charumanee S. Jiranusornkul S. Sirisa-Ard P. Sirithunyalug B. Sirithunyalug J. (2020). Depletion of β-sitosterol and enrichment of quercetin and rutin in Cissus quadrangularis Linn fraction enhanced osteogenic but reduced osteoclastogenic marker expression. BMC Complement. Med. Ther. 20 (1), 105. 10.1186/s12906-020-02892-w 32245457 PMC7119164

[B32] Saul D. Kosinsky R. (2021). Epigenetics of aging and aging-associated diseases. Int. J. Mol. Sci. 22 (1), E401. 10.3390/ijms22010401 33401659 PMC7794926

[B33] Shannon P. Markiel A. Ozier O. Baliga N. Wang J. Ramage D. (2003). Cytoscape: A software environment for integrated models of biomolecular interaction networks. Genome Res. 13 (11), 2498–2504. 10.1101/gr.1239303 14597658 PMC403769

[B34] Shen Z. Chen Z. Li Z. Zhang Y. Jiang T. Lin H. (2020). Total flavonoids of rhizoma drynariae enhances angiogenic-osteogenic coupling during distraction osteogenesis by promoting type H vessel formation through PDGF-BB/PDGFR-β instead of HIF-1α/VEGF Axis. Front. Pharmacol. 11, 503524. 10.3389/fphar.2020.503524 33328980 PMC7729076

[B35] Sheng S. Yang Z. Xu F. Huang Y. (2020). Network pharmacology-based exploration of synergistic mechanism of guanxin II formula (II) for coronary heart disease. Chin. J. Integr. Med. 27, 106–114. 10.1007/s11655-020-3199-z 32388823

[B36] Shuai Y. Jiang Z. Yuan Q. Tu S. Zeng F. (2020). Deciphering the underlying mechanism of eucommiae cortex against osteoporotic fracture by network pharmacology. Evid. Based. Complement. Altern. Med. 2020, 7049812. 10.1155/2020/7049812 PMC7492876 32963568

[B37] Stelzer G. Rosen N. Plaschkes I. Zimmerman S. Twik M. Fishilevich S. (2016). The GeneCards suite: From gene data mining to disease genome sequence analyses. Curr. Protoc. Bioinforma. 541, 1–30. 10.1002/cpbi.5 27322403

[B38] Szklarczyk D. Morris J. Cook H. Kuhn M. Wyder S. Simonovic M. (2017). The STRING database in 2017: Quality-controlled protein-protein association networks, made broadly accessible. Nucleic Acids Res. 45, D362–D368. 10.1093/nar/gkw937 27924014 PMC5210637

[B39] Tang W. Yang F. Li Y. de Crombrugghe B. Jiao H. Xiao G. (2012). Transcriptional regulation of vascular endothelial growth factor (VEGF) by osteoblast-specific transcription factor osterix (osx) in osteoblasts. J. Biol. Chem. 287, 1671–1678. 10.1074/jbc.M111.288472 22110141 PMC3265850

[B40] Trott O. Olson A. (2010). AutoDock Vina: Improving the speed and accuracy of docking with a new scoring function, efficient optimization, and multithreading. J. Comput. Chem. 31 (2), 455–461. 10.1002/jcc.21334 19499576 PMC3041641

[B41] Tsaioun K. Blaauboer B. Hartung T. (2016). Evidence-based absorption, distribution, metabolism, excretion (ADME) and its interplay with alternative toxicity methods. ALTEX 33 (4), 343–358. 10.14573/altex.1610101 27806179

[B42] UniProt Consortium T. (2018). UniProt: The universal protein knowledgebase. Nucleic Acids Res. 46 (5), 2699. 10.1093/nar/gky092 29425356 PMC5861450

[B43] Wang X. Wang N. Zhang Y. Gao H. Pang W. Wong M. (2008). Effects of eleven flavonoids from the osteoprotective fraction of Drynaria fortunei (KUNZE) J. SM. on osteoblastic proliferation using an osteoblast-like cell line. Chem. Pharm. Bull. 56 (1), 46–51. 10.1248/cpb.56.46 18175973

[B44] Wang Y. Zhang S. Li F. Zhou Y. Zhang Y. Wang Z. (2020). Therapeutic target database 2020: Enriched resource for facilitating research and early development of targeted therapeutics. Nucleic Acids Res. 48, D1031–D1041. 10.1093/nar/gkz981 31691823 PMC7145558

[B45] Wei X. Xu A. Shen H. Xie Y. (2017). Qianggu capsule for the treatment of primary osteoporosis: Evidence from a Chinese patent medicine. BMC Complement. Altern. Med. 17 (1), 108. 10.1186/s12906-017-1617-3 28193278 PMC5307793

[B46] Wong R. Rabie B. Bendeus M. Hägg U. (2007). The effects of rhizoma curculiginis and rhizoma drynariae extracts on bones. Chin. Med. 2, 13. 10.1186/1749-8546-2-13 18093297 PMC2206024

[B47] Wu Z. Zhu X. Xu C. Chen Y. Zhang L. Zhang C. (2017). Effect of Xianling Gubao capsules on bone mineral density in osteoporosis patients. J. Biol. Regul. Homeost. Agents 31 (2), 359–363. 28685537

[B48] Xiao W. Sun W. Lian H. Shen J. (2020). Integrated network and experimental pharmacology for deciphering the medicinal substances and multiple mechanisms of duhuo jisheng decoction in osteoarthritis therapy. Evid. Based. Complement. Altern. Med. 2020, 7275057. 10.1155/2020/7275057 PMC7657680 33204290

[B49] Xiong Z. Zheng C. Chang Y. Liu K. Shu L. Zhang C. (2021). Exploring the pharmacological mechanism of duhuo jisheng decoction in treating osteoporosis based on network pharmacology. Evidence-based complementary Altern. Med. 5510290, 2021–21. 10.1155/2021/5510290 PMC8046540 33880122

[B50] Xu C. Li R. Wu J. (2020). Effects of Yuanhu- Zhitong tablets on alcohol-induced conditioned place preference in mice. Biomed. Pharmacother. = Biomedecine Pharmacother. 133, 110962. 10.1016/j.biopha.2020.110962 33166765

[B51] Yu G. Wang L. Han Y. He Q. (2012). clusterProfiler: an R package for comparing biological themes among gene clusters. Omics a J. Integr. Biol. 16 (5), 284–287. 10.1089/omi.2011.0118 PMC3339379 22455463

[B52] Zhang C. Cho K. Huang Y. Lyons J. P. Zhou X. Sinha K. (2008). Inhibition of Wnt signaling by the osteoblast-specific transcription factor Osterix. Proc. Natl. Acad. Sci. U. S. A. 105, 6936–6941. 10.1073/pnas.0710831105 18458345 PMC2383965

[B53] Zhang Y. Jiang J. Shen H. Chai Y. Wei X. Xie Y. (2017). Total flavonoids from rhizoma drynariae (gusuibu) for treating osteoporotic fractures: Implication in clinical practice. Drug Des. devel. Ther. 11–1890. 10.2147/DDDT.S139804 PMC5491704 28694688

[B54] Zheng H. Feng H. Zhang W. Han Y. Zhao W. (2020). Targeting autophagy by natural product Ursolic acid for prevention and treatment of osteoporosis. Toxicol. Appl. Pharmacol. 115271, 115271. 10.1016/j.taap.2020.115271 33065153

